# Dynamic movement and turnover of extracellular matrices during tissue development and maintenance

**DOI:** 10.1080/19336934.2022.2076539

**Published:** 2022-07-20

**Authors:** Yutaka Matsubayashi

**Affiliations:** Department of Life and Environmental Sciences, Bournemouth University, Talbot Campus, Dorset, Poole, Dorset, UK

**Keywords:** Extracellular matrix (ECM), ECM movement, ECM turnover, morphogenesis, tissue maintenance

## Abstract

Extracellular matrices (ECMs) are essential for the architecture and function of animal tissues. ECMs have been thought to be highly stable structures; however, too much stability of ECMs would hamper tissue remodelling required for organ development and maintenance. Regarding this conundrum, this article reviews multiple lines of evidence that ECMs are in fact rapidly moving and replacing components in diverse organisms including hydra, worms, flies, and vertebrates. Also discussed are how cells behave on/in such dynamic ECMs, how ECM dynamics contributes to embryogenesis and adult tissue homoeostasis, and what molecular mechanisms exist behind the dynamics. In addition, it is highlighted how cutting-edge technologies such as genome engineering, live imaging, and mathematical modelling have contributed to reveal the previously invisible dynamics of ECMs. The idea that ECMs are unchanging is to be changed, and ECM dynamics is emerging as a hitherto unrecognized critical factor for tissue development and maintenance.

## 1. Introduction

### 1.1. Background

The body of multicellular animals is made of not only cells but also extracellular matrices (ECMs) that consist of many different proteins and polysaccharides. ECMs provide structural integrity to various body parts such as the skin, tendon, and bone; ECMs also work as scaffolds on/in which cells reside, divide, differentiate, and migrate; moreover, ECMs regulate signal transduction by directly activating matrix receptor molecules such as integrin, or by working as a reservoir of growth factors and cytokines. Deficiencies in these architectural and signalling functions can cause severe tissue defects and/or diseases (see section 1.2 below).

While acknowledged to play various essential roles, ECMs have often been thought of as stable structures with little or no movement or replacement of components. This idea seemed to be supported by some early studies reporting that ECM components have long half-lives on the order of months or more [1,2]. Consequently, in the early 1980s ECMs were even compared to ‘the styrofoam packing material’, which merely ‘“fills the spaces” between cells and tissues’ [3]. However, if ECMs are so stable, how can tissues containing them grow and change shape during development? Moreover, many ECMs, e.g. those in tendons, bones, and cartilages, experience repeated mechanical loads; how can these ECMs avoid fatigue failure if their components are not replaced? Regarding these conundrums, this article reviews multiple lines of evidence that ECMs are in fact rapidly moving and turning over in various animals. The mechanisms and functional importance of the movement and turnover are also discussed. The review begins with brief primers on the representative ECMs (section 1.2) and the concepts of ECM ‘movement’ and ‘turnover’ (section 1.3).

### 1.2. The structures, components, and functions of representative ECMs

**Figure 1.**
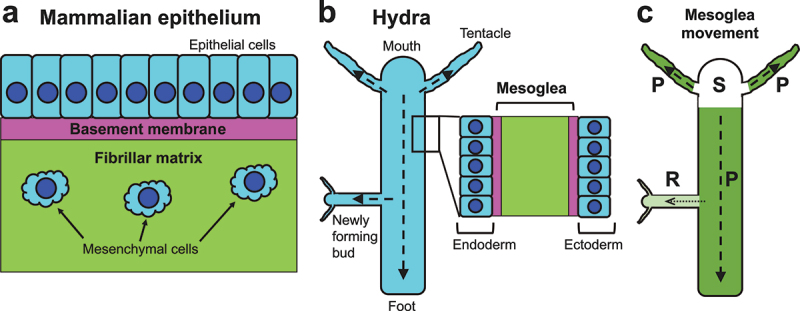
Schematics of the three extracellular matrices (ECMs) (a and b), and the movement of hydra epithelia and mesogloea (b and c). (a) The basement membrane and fibrillar matrix in a mammalian epithelial tissue. Epithelial cells reside on the flat sheet of the basement membrane (magenta), while mesenchymal cells are dispersed in the three-dimensional fibrillar matrix (green). (b) Mesogloea in the body of a hydra. The area inside the rectangle in the left image is enlarged in the right. The body wall is made of two sheets of epithelia: the endoderm facing the gut lumen and the ectoderm facing outside. Between the two epithelia there exists an ECM called mesogloea. The mesogloea consists of basement membrane-like (magenta) and fibrillar matrix-like (green) regions. Dashed arrows show the direction of epithelial cell movement discussed in section 2.6. (c) The movement of mesogloea (section 2.6). S (white), mesogloea is Stationary in the head; P (dark green), mesogloea moves in Parallel with the migration of epithelia shown in (b); R (light green), a Restricted amount of mother’s mesogloea moves into daughters.

ECMs are formed at both the apical and basal sides of epithelia, i.e. outside and inside the body, respectively. At the apical side, ECMs such as the mucin-based mucus layer of vertebrates or the chitin-based cuticle of insects and other invertebrates cover, protect, and shape the body surface [4–8]. At the basal side, there also exist ECMs made of different components such as collagen and fibronectin (see below). Although it is interesting to discuss the dynamics of each different matrix, for the sake of conciseness this article focuses on three basal ECMs that are relatively more studied: the basement membrane, fibrillar matrix, and hydra mesogloea (Figure 1). Their structures, components, and functions are briefly overviewed below.

The basement membrane is a thin layer of ECM (Figure 1a) that exists in almost all multicellular animals [9,10]. This flat matrix underlies all the epithelia; moreover, the basement membrane also wraps muscles, nerves, and adipose tissues to separate them from neighbouring structures. Defects in the basement membrane functions cause various problems such as skin blistering (when the basement membrane under the epidermis is affected) or proteinuria (when the basement membrane in the renal glomeruli is deficient). All the basement membranes contain a common set of evolutionarily conserved components: laminin, type IV collagen, nidogen, and the heparan sulphate proteoglycan perlecan and/or agrin. Networks of laminin and type IV collagen form the core platform of the basement membrane, to which the glycoprotein nidogen and the large proteoglycan perlecan bind. In addition to these core components, the basement membrane also contains various other proteins, glycoproteins, and proteoglycans. Basement membranes in different tissues harbour distinct sets of components; this component diversity is supposed to enable each basement membrane to have unique biochemical and biophysical properties, and to play location-specific roles such as the determination of stem cell niches [11–21]. Various cell types are involved in basement membrane formation. For example, in the mouse skin, keratinocytes and fibroblasts cooperate to construct the basement membrane by producing different components such as laminin and type IV collagen [15]. In *Drosophila*, haemocytes (macrophages), fat body cells, and egg follicle cells express type IV collagen. While haemocytes and follicle cells locally deposit type IV collagen to construct the basement membrane on nearby tissues [22–26], fat body cells secrete type IV collagen into the haemolymph (blood), which deliver the secreted protein to distant organs such as the imaginal discs [27].

While the basement membranes are flat sheets, fibrillar matrices (also called interstitial matrices) form three-dimensional structures (Figure 1a). Fibrillar matrices are especially prominent and well-studied in vertebrates; these matrices provide integrity, elasticity, and rigidity to various body parts such as the skin, tendon, and bone. Major components of vertebrate fibrillar matrices include fibrillar collagens (types I, II, III, V, XI, XXIV and XXVII [28]), elastin, and fibronectin. Fibroblasts have been well known as a main source of these components [28,29]; however, recent studies are revealing that macrophages also produce a significant amount [30–35]. Loss of fibrillar matrix components causes tissue fragility as seen in diseases such as osteoporosis and arthritis; in contrast, excess production leads to fibrosis such as scars, keloids, and cirrhosis [3,18,28,36–40]. Fibrillar matrix components are not necessarily universal in the Animal Kingdom: for example, invertebrates do not produce fibronectin; *Drosophila* lack fibrillar collagens, which exist in bees, mosquitoes, and many other animals [9,41–43].

Mesogloea is the ECM of hydra, one of the most primitive multicellular animals. The body of hydra is organized as a gastric tube with a mouth surrounded by tentacles at the top and a foot process at the bottom (Figure 1b). The body wall is made of two layers of epithelia (ectoderm and endoderm); between the layers there exists the ECM mesogloea, which supports proper cell proliferation, migration, and differentiation. Mesogloea is composed of both basement membrane- and fibrillar matrix-like structures: adjacent to each epithelial layer there is a defined basement membrane-like region containing laminin and type IV collagen; between the two basement membrane-like regions there exists a fibrillar matrix-like domain containing collagen I and presumably other fibrillar collagens as well (Figure 1b) [44,45].

ECMs play not only architectural but also signalling roles. For example, the basement membrane and fibrillar matrix can directly activate their receptors such as integrin, dystroglycan, and syndecan, which regulate various cellular responses including gene expression. Moreover, the two ECMs can also modulate cellular signalling indirectly, by acting as a reservoir for secreted signalling molecules such as fibroblast growth factors and transforming growth factor-β: matrix binding of these factors can either promote or restrict ligand-receptor interactions. Failure in the direct/indirect signalling by the basement membrane or fibrillar matrix are suggested to cause various defects such as the abnormal morphogenesis of the heart, lungs, and limbs, and failures in glial cell migration and mammary epithelial cell differentiation [3,11,36,46]. As to hydra mesogloea, its signalling functions are yet to be studied. However, hydra genome contains integrin and integrin-associated signalling molecules such as the focal adhesion kinase and the integrin-linked kinase [47]. Moreover, it has been reported that a mesogloea protein HmTSP (hydra thrombospondin) may be modulating Wnt signalling [48]. These results suggest that mesogloea also plays signalling roles as well as the basement membrane and fibrillar matrix do. The following sections review the movement and turnover of these three ECMs.

### 1.3. Concepts of ECM ‘movement’ and ‘turnover’

First, this section clarifies what ‘movement’ and ‘turnover’ mean in this article.

**Figure 2.**
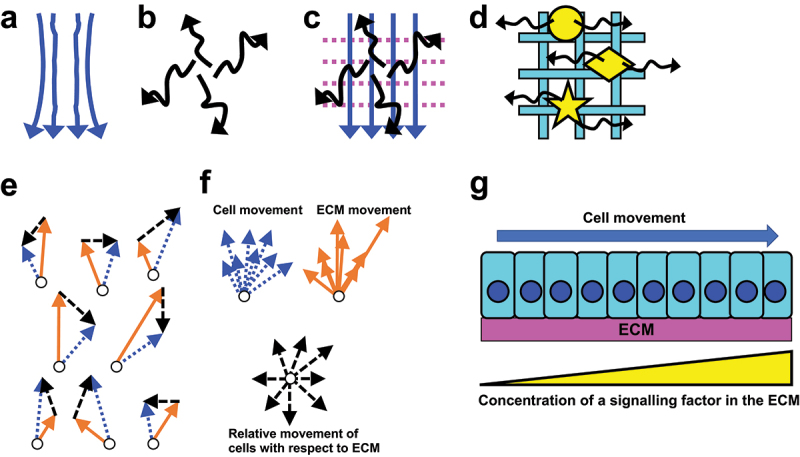
Movement of ECMs and cells. (a and b) Potential movements of ECM components: (a) directional displacement and (b) non-directional migration. (c) Co-existence of hypothetical components that are moving directionally (blue arrows), non-directionally (black arrows), and staying stationary (magenta dots). (d) Model of the basement membrane of *C. elegans* [17]. Mobile components such as fibulin, agrin, and nidogen (yellow circle, diamond, and star) are moving bi-directionally within a stable scaffold containing laminin and type IV collagen (cyan lattice). (e and f) Relative movement of cells with respect to ECM. (e) In a hypothetical tissue, both cells (open circles) and the ECM associated with them (not depicted) are moving towards the top of the image. Blue dotted arrows show the velocity of each cell, and Orange solid arrows show the velocity of the ECM at the position of each cell. The vector differences between the blue and Orange arrows of each cell (black dashed arrows) show the relative motion of the cell with respect to the ECM. (f) The arrows of each colour in (e) are gathered so that they initiate from a single point. The blue and Orange arrows are aligned to the top of the image, reflecting that both the cells and ECM are moving upward when seen from outside the tissue. In contrast, black arrows are not aligned, indicating that cells are moving towards any direction in the reference frame fixed on the moving ECM. (g) ECM movement can affect cell-ECM signalling. In a hypothetical tissue, a group of cells are moving on an ECM that contains a gradient of a signalling molecule (yellow triangle). The arrow shows the direction of cell movement seen from outside the tissue. If the ECM is stationary, the cells are moving towards a higher concentration of the signalling molecule. In contrast, if the ECM is moving together with the cells, each cell is exposed to a constant level of the signal during the cell-ECM co-migration.

The word ‘movement’ can be used to describe many different phenomena. In this article, the word refers either to directional displacement like e.g. water flow (Figure 2a) or non-directional migration whose examples include the random walk of particles [49] (Figure 2b). Theoretically, ECM components that are carrying out different movements and/or those moving at different speeds can coexist in one place (Figure 2c). Section 2 below reviews the actual cases of ECM movements.

‘Turnover’ is the cycle of component neosynthesis and degradation; net changes in the amount of ECM components are determined by the balance between synthesis and degradation. Section 3 discusses the baseline turnover of ECMs both in developing and mature tissues.

## 2. Movement of ECMs

### 2.1. Dawn of ECM movement research in the 1980s

In 2020, two papers reporting the rapid movement and turnover of basement membrane components were published [17,50]. In response to these papers, a preview article commented that ‘the idea that the basement membrane is a stable and unchanging structure is being turned over’ [51]. This indicates that papers reporting ECM dynamics were still regarded surprising in 2020. However, in the 1980s, around the time when ECMs were called ‘the styrofoam packing material’ (section 1.1) [3], some experiments had already started to shed light on ECM movements. This and following sections show two examples of such early reports, and how subsequent studies using more modern technologies followed them up, finally ‘turning over’ the idea of stable ECMs.

**Figure 3.**
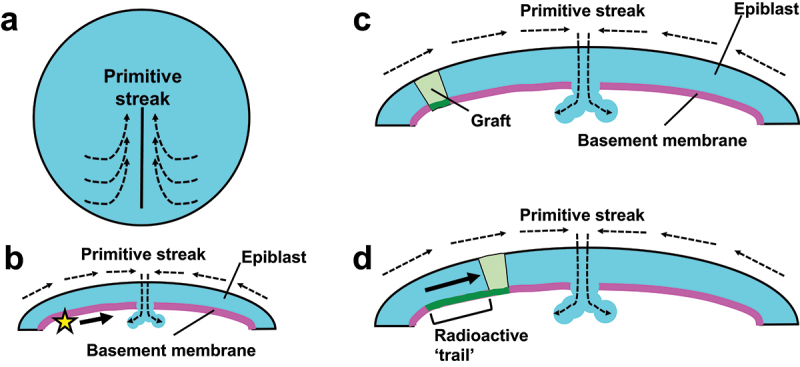
Basement membrane movement in the early avian embryo. (a and b) Schematics of basement membrane movement in the gastrulating avian embryo at HH stage 2. Dashed arrows show the direction of cell migration. (a) Top view. The epiblast cells perform a swirling collective migration towards the primitive streak (black solid line). (b) Cross section. A basement membrane (magenta) underlies the epiblast (cyan). Epiblast cells ingress inside the embryo at the primitive streak, where the basement membrane is degraded. When a basement membrane-labelling marker was applied at the position of the star, the dye spread to the direction of the primitive streak (solid arrow) [54]. (c and d) Relative movement of avian epiblast cells with respect to the basement membrane during gastrulation. (c) A fragment of quail epiblast (green) was loaded with radioactive glucosamine and grafted into the epiblast of a chick embryo. Dashed arrows show the direction of cell migration as in (b). (d) 6 hours later, grafted cells moved closer to the primitive streak (thick solid arrow), leaving a ‘trail’ of radioactivity in the basement membrane (thick green line).

*Basement membrane movement in the early avian embryo*. The early avian embryo has a flat disc-like structure; the outermost layer of the disc is covered by a one-cell thick epithelium called the epiblast. During gastrulation, which starts several hours after egg laying, or at Hamburger and Hamilton (HH) stage 2 in the term used in avian developmental biology [52], the epiblast cells undergo a swirling collective migration towards the primitive streak, where the epiblast cells ingress inside the embryo to form the mesoderm and endoderm (Figure 3a) [53]. Underneath the epiblast, there exists a basement membrane (Figure 3b). In 1984, it was examined whether this basement membrane stays stationary or moves with the epiblast during gastrulation. A basement membrane-labelling marker concanavalin A-ferritin conjugate was topically applied to the basement membrane under the epiblast of a cultured chick gastrula, on a spot that is located ~500 µm laterally to the primitive streak (Figure 3b). After 5 hours incubation, the label was found to have spread medially towards the primitive streak but not to the opposite direction; this spreading occurred at 37°C but not at 4°C. From these results, it was proposed that the basement membrane is moving actively and directionally (see Figure 2a) towards the primitive streak, in the same direction as the migration of epiblast cells [54]. Based on the distance over which the dye spread (~500 µm) and incubation time (5 hours), the speed of the proposed basement membrane movement can roughly be estimated as ~100 µm/hour (Table 1).

**Table 1.** The speeds of the ECM movements mentioned in this review.

Episode	Probe	Approx. speed (µm/hour)	Section	Ref
Basement membrane movement under the avian epiblast	ConA-ferritin conjugate	100	2.1	[54]
Basement membrane movement under the avian epiblast	Anti-fibronectin Ab	10–50	2.6	[62]
Basement membrane movement in the developing mouse salivary gland	Radioactive glucosamine	50	2.1	[56]
Basement membrane movement in the developing mouse salivary gland	Anti-type IV collagen Ab	8	2.3	[64]
Fibrillar matrix movement in the early avian embryo	Anti-fibrillin Ab	30	2.2	[57]
Fibrillar matrix movement in the early avian embryo	Anti-fibronectin Ab	20–40	2.2	[58]
Mesogloea movement in hydra:	Anti-collagen-1 Ab		2.6	[63]
In the body column		4		
In tentacles		10		
Basement membrane component movement in the *C. elegans* pharynx:	mNeonGreen		2.5	[17]
fibulin		610 ± 100 (*)		
peroxidasin-1		680 ± 70 (*)		
agrin		290 ± 70 (*)		
nidogen		220 ± 70 (*)		
spondin		320 ± 40 (*)		

If the speed was not explicitly described in the refs, a rough estimation based on representative images or text was carried out. Ab, antibody; ConA, concanavalin A. Antibodies were labelled with fluorescence. (*) Mean ± standard deviation, n = 5.

**Figure 4.**
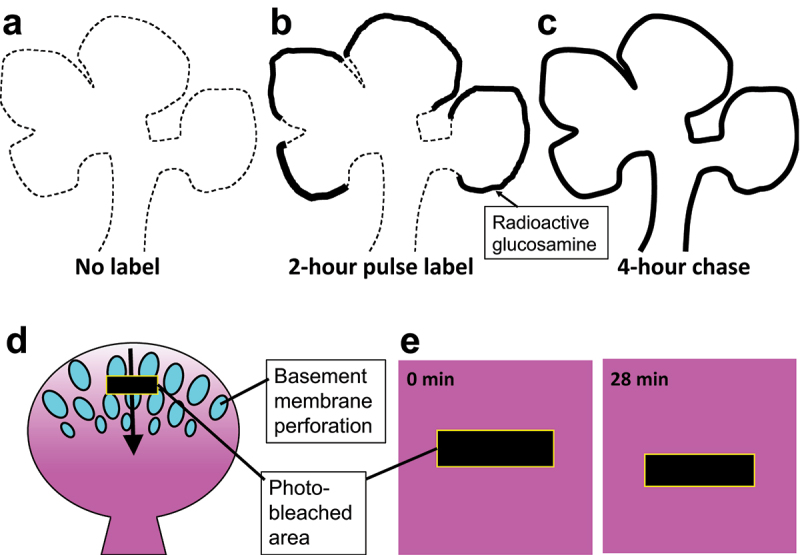
Basement membrane movement during the epithelial branching of the mouse submandibular salivary gland. (a-c) Schematics of a classical pulse-chase experiment that suggested the movement of basement membrane components [56]. (a) Dashed line indicates the contour of the growing epithelium in the developing salivary gland cultured *ex vivo*. (b) The tissue was incubated for 2 hours with radioactive glucosamine, a building block of basement membrane glycosaminoglycans. The radioactivity was incorporated into the distal parts of the epithelial buds (solid lines). (c) The tissue was subsequently chased in non-radioactive medium for 4 hours. The radioactivity spread to the base of the buds. (d and e) Live imaging visualizes the perforation and movement of the basement membrane [64]. (d) Epithelial buds in the growing salivary gland were labelled with a fluorescent anti-type IV collagen antibody. At the top of the buds, the basement membrane was perforated by numerous holes (illustrated as ellipses) through which the epithelium (cyan inside the ellipses) directly interacted with the fibrillar matrix (not depicted) outside the basement membrane. The basement membrane moved from the top to the bottom of the bud (arrow), making the matrix denser in the lower part of the bud (symbolized by the gradient of magenta colour). When a rectangular region (black) was photobleached, the rectangle moved towards the bottom of the bud as shown in the enlarged images in (e). Note that the matrix perforation and type IV collagen gradient are not depicted in (e) for the sake of simplicity.

*Basement membrane component movement during epithelial branching morphogenesis*. Organs such as the lung, kidney, and salivary gland have highly branched networks of epithelial tubules, maximizing surface area for secretion and absorption. During the development of these organs, epithelial tubules arise as rounded buds, undergo repetitive branching and outgrowth, and eventually form a highly multifurcated network (Figure 4a). Throughout this process, the growing epithelial network is always surrounded by the basement membrane [55]. Therefore, it seems likely that the basement membrane is also undergoing a dynamic remodelling to accommodate the drastic outgrowth of the epithelial network. Indeed, a classical work [56] suggested that basement membrane components are moving from the tip to the bottom of growing epithelial buds. Mouse submandibular salivary glands were cultured *ex vivo* and labelled with radioactive glucosamine that was to be incorporated into the glycosaminoglycans in the basement membrane; subsequently the glands were washed with and incubated in non-radioactive medium. This pulse-chase experiment showed that the radioactivity was first incorporated into the basement membrane located at the distal parts of the epithelial buds, and then moved to the basement membrane covering the base of the buds (Figure 4a-c). In the images presented in the paper, the radioactive probe seemed to have spread ~100 µm in 2 hours. Thus, the migration speed of glucosamine-containing basement membrane components can be roughly estimated as ~50 µm/hour (Table 1). There are at least two possible interpretations about the net movement of incorporated glycosaminoglycan: 1) each single molecule directionally moved from the tip to the bottom of the buds (see Figure 2a), and 2) the molecules are incorporated into the matrix only at the tip, then spread to the bottom by random walk (see Figure 2b). It cannot be distinguished which was the case.

### 2.2. Fluorescent antibodies allow for live imaging of ECM movement

Years later, it became possible to live-image ECMs by labelling their components with fluorescently tagged antibodies. First, a vortex-like, global and directional movement of ECMs containing fibrillin [57] or fibronectin [58] was discovered in the avian embryo at HH stages 5–10, when the notochord and somites are being formed. At this stage, while fibrillin is only in the interstitial fibrillar matrix [57,59], fibronectin exists both in the fibrillar matrix and the basement membrane [58,60,61]. Thus, the fibronectin antibody seems to have detected the average movement of multiple ECMs; it would be interesting to also examine the movement of basement membrane-specific markers such as type IV collagen and laminin.

Antibody-based live imaging was also used to examine many other ECM movements: the classical report of the basement membrane movement during avian gastrulation (section 2.1) [54] was recently supported by a new study [62]; hydra mesogloea was found to be moving in the body of this simple animal [63]. Details of these work will be discussed later (section 2.6). Moreover, as reviewed in the next section, the basement membrane movement in the developing mouse submandibular salivary gland (section 2.1) [56] was revisited and investigated further.

### 2.3. Live imaging confirms the basement membrane movement during epithelial branching, and partially reveals its molecular mechanism and functional importance

Visualization of the basement membrane of the developing mouse submandibular salivary gland with non-perturbing type IV collagen antibody [64] revealed that the basement membrane was perforated by hundreds of microscopic holes at the tip of the newly forming epithelial bud, and that the perforated basement membrane moved from the top to the bottom of the bud at the average speed of 8 µm/hour (Figure 4d, e; Table 1). When a rectangular region was photobleached, the bleached rectangle moved parallelly, largely keeping the shape (Figure 4e). This suggests that a stable network of collagen IV molecules is coherently carrying out a directional migration (see Figure 2a). The direction of basement membrane movement was consistent with the result of the previous pulse-chase experiment (section 2.1; Figure 4a-c)[56]. The newly obtained migration speed (8 µm/hour) seems slower than that suggested from the classical research (~50 µm/hour); however, because the latter value is based on a very rough estimation, the two speeds might be considered consistent. Alternatively, the glucosamine-labelled basement membrane component(s) in the pulse-chase experiment may have moved more quickly than type IV collagen. A study in. *Caenorhabditis elegans* showed that different basement membrane components are moving at different speeds and that type IV collagen is one of the slowest moving proteins [17] (see Figure 2c and section 2.4 below).

Subsequently, the mechanism and function of the basement membrane movement were examined. A broad specificity metalloprotease inhibitor batimastat/BB-94 and a myosin II inhibitor blebbistatin were found to block the basement membrane movement, perforation, and epithelial branching. Basement membrane perforation was also inhibited by Y27632, which indirectly reduces myosin II activity by blocking its activator ROCK [65]. Compared to batimastat, more specific protease inhibitors showed less or no effects, suggesting that multiple proteases are involved in the basement membrane dynamics and branching morphogenesis. From these results, it was suggested that proteolysis by multiple enzymes makes the basement membrane pliable, that myosin II-dependent cell forces move the softened basement membrane, and that these basement membrane pliability and movement help the growth of newly forming epithelial buds [64].

Recently, it was reported that in the early post-implantation mouse embryo, the basement membrane covering expanding epithelium is also perforated, and that proteolysis mediated by matrix metalloproteinases helps the basement membrane perforation and tissue expansion [66]. It would be interesting to test whether proteolysis-dependent basement membrane perforation is a general phenomenon occurring when tissues surrounded by the basement membrane expand, whether the basement membrane is also moving in the mouse embryo, and whether basement membrane movement and perforation are always associated with each other.

### 2.4. Basement membrane proteins move within scaffolds made by laminin and type IV collagen

In addition to fluorescent antibodies, genetically coded fluorescent proteins are also powerful tools for live imaging, especially when combined with a simple model organism and genome-engineering techniques. Recently, by utilizing CRISPR-Cas9-mediated homologous recombination, 17 basement membrane proteins in *C. elegans* were endogenously labelled with a green fluorescent protein mNeonGreen, with minimum perturbations of their functions [17]. This allowed for the comprehensive analysis of the dynamics of the labelled proteins *in vivo*. Surprisingly, fluorescence recovery after photobleaching (FRAP) assay revealed that many basement membrane components such as fibulin, agrin, and nidogen, were rapidly moving in the basement membrane surrounding the pharynx of L4-stage larvae. After photobleaching, ~30-65% of their original fluorescence was recovered within only 15 minutes.

**Figure 5.**
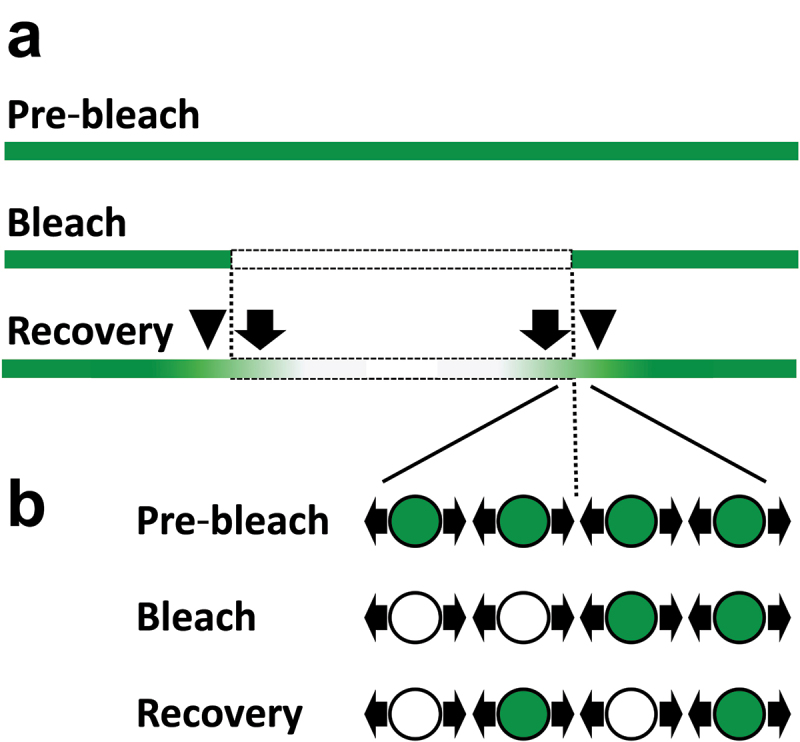
Bi-directional movement of *C. elegans* fibulin in the basement membrane. (a) Sagittal optical section of the basement membrane containing mNeonGreen-labelled fibulin (green). Top, before bleaching. Middle, central area (dashed rectangle) was photobleached. Bottom, recovery of fluorescence. While green fibulin spread into the bleached area (arrows), fluorescence intensity in the area adjacent to the bleached area decreased (arrowheads). (b) Model explaining the result. The region near the border of the bleached area is enlarged. Green and white circles show fibulin molecules with intact and bleached mNeonGreen, respectively. Each molecule is moving bidirectionally (arrows). After photobleaching, this bidirectional movement causes the exchange of fluorescent and non-fluorescent molecules, leading to the increase and decrease of green fluorescence inside and outside the bleached area, respectively.

The fluorescence recovery occurred from the edge of the bleached region, suggesting that basement membrane components are moving laterally from the adjacent unbleached area (Figure 5a, arrows). Each component moved 5 µm into the bleached region at the following rates (mean ± standard deviation, n = 5): fibulin (0.17 ± 0.04 µm/s), peroxidasin-1 (0.19 ± 0.02 µm/s), agrin (0.08 ± 0.02 µm/s), nidogen (0.06 ± 0.02 µm/s), and spondin (0.09 ± 0.01 µm/s) (Table 1). As the fluorescence inside the bleached area recovered, the intensity at the unbleached area decreased, suggesting that the lateral fibulin movement was at least ‘bi’-directional (Figure 5a arrowheads and b) [17]. It is simple to hypothesize that the movement was ‘non’-directional (Figure 2b), e.g. fibulin was randomly diffusing in the matrix. However, because all the analysis was carried out only in one dimension using optical sections, the possibility that the movement was restricted to the directions parallel to the sections cannot be excluded completely.

Subsequently, it was suggested that the dynamic movement of basement membrane components was an active process, because treatment with an ATP synthesis inhibitor dicyclohexylcarbodiimide (DCCD) led to a ~ 70% decrease in the fluorescence recovery rate of fibulin. It was hypothesized that a possible ATP-dependent regulator of the movement might be muscle contractions, which could distribute energy into the basement membrane. Consistently, muscle-paralysing drugs 2,3-Butanedione monoxime (BDM), levamisole, or the mix of levamisole and tricaine caused ~50-70% reductions of the fibulin mobility [17]. However, it should be noted that off-target effects of the drugs cannot be excluded: for example, BDM is known to affect various proteins such as non-muscle myosins, gap junction connexins, and several ion channels [67]. Genetic perturbation of muscle contraction and other potential drug targets will be necessary to further understand the mechanisms of the matrix motility.

Interestingly, laminin and type IV collagen, which are known to make the core structure of the basement membrane (see section 1.2), showed much lower mobilities than fibulin and other mobile proteins. Moreover, not only in the pharynx basement membrane but also in the gonad basement membrane, nidogen was mobile and laminin was immobile, suggesting that the matrix movement and the difference in the mobility among components are general properties of the basement membrane. From these results, a model that dynamic basement membrane proteins are moving on the stable core scaffold made by laminin and type IV collagen was proposed (Figure 2d) [17].

### 2.5. ECMs can move as fast as cells do, both during tissue remodelling and in steady state

The classical and recent studies reviewed above show that ECMs are not stationary but in fact highly mobile, in diverse animals ranging from hydra to mammals. There were several different modes of ECM movement: for example, ECMs in the avian embryo showed vortex-like global movements (section 2.2; see also section 2.6); in the developing mouse salivary gland, it was suggested that a stable network of type IV collagen parallelly moved to one orientation (section 2.3); hydra mesogloea directionally displaced towards body peripheries as described in the section 2.6 below; in the basement membrane of *C. elegans*, fibulin was moving at least ‘bi’-directionally, or possibly ‘non’-directionally (section 2.4) within a stable matrix scaffold. The estimated speeds of ECM movement (about 4–700 µm/hour, Table 1) were comparable to the velocities of cell migration seen in various events occurring *in vivo* and *in vitro* (Table 2). Thus, ECMs can move as fast as cells do.

**Table 2.** Examples of cell migration speeds.

Episode	Approx. speed (µm/hour)	Refs
Confinement release assay of HaCaT cells	5–30	[149]
Scratch wound assay of MDCK cells	10–40	[150–152]
Random migration of B16-F1 melanoma cells cultured on fibronectin	0–60	[153]
Hair follicle invagination in the mouse embryo	4–8	[154]
Neural crest cell migration in the zebrafish or *Xenopus* embryo	20–90	[155, 156]
Macrophage/haemocyte movement during *Drosophila* embryogenesis	60–180	[157, 158]
Macrophage recruitment to wounds in the zebrafish embryo	300–500	[159, 160]

If the speed was not explicitly described in the refs, a rough estimation based on representative images was carried out.

ECM movement occurs both during tissue remodelling and in steady state: while the avian epiblast and mouse submandibular salivary gland (sections 2.1 and 2.3) were undergoing drastic structural changes, the pharynx of *C. elegans* L4 larvae (section 2.4) was no longer increasing its size or the levels of basement membrane components [17]. Another example of ECM movement in steady state is the mesogloea movement in the body column and tentacles of adult hydra discussed in the next section.

### 2.6. Relative movement of cells with respect to ECMs

When ECMs move, do cells residing on/in them move together? Hydra provides a powerful experimental system to address this question. It was known that hydra epithelial cells constantly move towards the periphery of the body and into outgrowing buds during homoeostasis and asexual reproduction by budding (Figure 1b). However, it was not known whether the mesogloea also moves in the body. To address this question, the cells and matrix were labelled by different colours using a live-cell dye and antibodies, respectively. Stained cells and matrix were grafted into another hydra together; subsequently their movement in the host body was observed. This analysis produced different results depending on the position of the labelled tissue (Figure 1c). First, in the body column and tentacles, epithelia and mesogloea moved together towards the foot and tentacle tips, respectively. The speed of mesogloea migration was coarsely estimated to be ~4 µm/hour in the body and ~10 µm/hour in tentacles. However, in the head, while mesogloea was immobile, epithelial cells left the stationary matrix and moved into tentacles. In the newly forming bud, while epithelial cells moved into the bud from the mother body, only a small amount of the mother hydra’s mesogloea moved into the growing daughter [63].

Relative movement of cells with respect to ECM was also analysed in the gastrulating avian embryo. While the epiblast and its basement membrane were suggested to be moving towards the same direction (section 2.1) [54], it was not known whether the epithelium and the matrix are completely moving together. To address this question, an epiblast fragment was taken from a quail embryo and labelled with radioactive glucosamine, a building block of basement membrane components. Subsequently, the fragment was grafted into a chicken embryo, in which the quail cells carried out gastrulation movement together with the host cells. If the epiblast and the basement membrane underneath it are moving together, the radioactive glucosamine should be deposited only to the matrix immediately below the grafted cells. However, in fact a radioactivity ‘trail’ extending from the labelled cells were formed in the basement membrane, suggesting that epiblast cells are moving relatively to the basement membrane (Figure 3c, d) [68].

Later, a more quantitative analysis was carried out by simultaneously live-imaging epiblast cells expressing GFP and the ‘basement membrane’ underneath them labelled by anti-fibronectin antibody [62]. In the HH stage 2 embryos used in this study, the fibronectin antibody labelled a thin sheet of ECM, but not a structure that looked like the fibrillar matrix. Thus, the movement of the antibody should have largely reflected the movement of the basement membrane. This experiment revealed global velocity fields of epiblast cells (cf. Figure 3a) and fibronectin, respectively (speed 10–50 µm/hour; Table 1). The two velocity fields were largely parallel to each other, but not completely identical, i.e. the cells were moving relatively to the matrix. This is consistent with the result of the grafting experiment mentioned in the previous paragraph [68]. The relative movement of the cells with respect to the matrix was calculated by subtracting the matrix velocity from the cell velocity (Figure 2e, f), and it was revealed that epiblast cells were undergoing a random walk on the moving matrix [62]. Using a rough metaphor based only on their relative motion, the ECM and cells can be compared to a ship and its passengers, respectively. Seen from outside, both the ship and passengers are heading towards the destination of the ship; but inside the ship, the passengers are walking towards any directions (Figure 2e, f).

Subsequently, the relative motility of mesoderm cells with respect to an ECM labelled with fibronectin antibody was also examined using older avian embryos (HH10-11) in which somites were being formed and the body axis was elongating. In this experiment, the fibronectin antibody predominantly labelled the interstitial fibrillar matrix. The results suggested that the cells were undergoing random movement in the reference frame fixed on the matrix [69]. Thus, in the avian embryo, mesenchymal cells in the mesoderm move randomly with respect to the fibrillar matrix, while epithelial cells in the epiblast move randomly with respect to the basement membrane.

In summary, these results obtained from hydra and birds show that the relative motion of cells with respect to moving matrix differs depending on context. It remains to be revealed what generates the force to move the co-migrating cells and ECMs. Are ECMs passively carried by cells, or can ECM elasticity, plasticity, and dynamic component turnover (see the following section 3) generate forces to move cells and tissues? Further discussion is in section 4.2.

## 3. ECM Turnover

### 3.1. Challenges to analyse baseline ECM turnover

While the movement of ECMs was reviewed above, the following sections discuss ECM turnover, i.e. the cycle of ECM component degradation and neosynthesis. ECM degradation is involved in many different physiological and pathological events including cancer, arthritis, tissue regeneration, and amphibian metamorphosis [70]. Therefore, the mechanisms of ECM breakdown have been extensively studied. Since the first report of a collagenolytic activity in frog tissues in 1962 [71], lots of ECM-digesting enzymes such as matrix metalloproteinases (MMPs), a disintegrin and metalloproteinases (ADAMs), and secreted cathepsins have been identified and investigated. Their structures, substrates, regulation mechanisms, and functions are thoroughly reviewed in other articles [39,40,70,72–77].

The studies of ECM degradation mentioned above were mainly done by analysing events involving the acute breakdown of ECMs, e.g. tadpole tail resorption and cancer invasion. However, net changes in the amount of ECM components are determined by the balance between synthesis and degradation. When tissues like the tadpole tail are being deconstructed, ECM degradation is readily observed while continuing ECM neosynthesis is obscured [78]. In contrast, when tissues are growing or in steady state, it is not obvious whether there is a baseline turnover of ECMs. Nevertheless, as reviewed in the following sections, recent studies are revealing that ECMs are rapidly turning over both during embryogenesis and in adult tissues.

### 3.2. *Mathematical modelling reveals a rapid basement membrane turnover in the* Drosophila *embryo*

While proteolysis was suggested to play important roles in basement membrane remodelling during embryogenesis (section 2.3) [64,66], it had not been quantified exactly how much overall degradation/turnover of the basement membrane is occurring during tissue development. Previous studies in postnatal animals reported a range of possible basement membrane turnover rates from hours to months (Table 3) [2,79–81]. However, considering that tissue development and remodelling occur in the time scale of hours, it could be plausible to suspect that the basement membrane in the embryo is also rapidly turning over within several hours.

**Table 3:** Reported half-lives of basement membrane components.

Animal	Age or BW	Tissue	Component(s) examined	Method	Externally added Probe	Probe delivery method	Half-life	Ref
Rat	>16 weeks	Kidney	Entire GBM	Pulse-chase	AgSO_4_ (*)	Mixed in drinking fluid	On the order of months or years	[1]
Rat	100-115g	Kidney	Entire GBM	Pulse-chase	^3^H glycine, ^3^H proline	i.p. injection	>70 days	[2]
Rat	100-115g	Kidney	Entire GBM	Pulse-chase	^3^H leucine, ^3^H lysine, ^3^H phenylalanine	i.p. injection	16-45 days	[2]
Rat	230-250g	Kidney	Collagen portion of the GBM	Pulse-chase	^3^H proline	i.p. injection	15-17 days	[161]
Rat	300g	Kidney	Entire GBM	Pulse-chase	^3^H glycine, ^3^H proline, ^14^C proline	i.p. injection	> 10 days	[80]
Rat	Not specified	Kidney	GAGs in the GBM	Pulse-chase	^35^S sulphate	i.p. injection	~7 days	[162]
Rat	Not specified	Kidney	Entire GBM	Pulse-chase	^35^S sulphate	i.p. injection	1 – several days	[163]
Human	0-85 years	Kidney	Entire GBM	Glycation (**)	NA	NA	4.5 days	[81]
Rat	180g	Kidney	HSPGs in the GBM	Pulse-chase	^35^S sulphate	i.p. injection	5-20 hours	[79]
Mouse	Adult, 22-26g	Gut	Laminin	Pulse-chase	Anti-laminin antibody	i.v. injection	On the order of weeks	[164]
Rat	160-200g	Cultured renal glomeruli	HSPGs	Pulse-chase	^35^S sulphate	Added to medium	11-47 hours	[165]
Rat	160g	Kidney	Perlecan in the GBM	Pulse-chase	^35^S sulphate	s.c. injection	3–4 hours	[166]
Mouse	6-8 weeks	Lung	α3 laminin	Inducible knockout	NA	NA	30-60 days (***)	[167]
*Drosophila*	Embryo	Whole body	Type IV collagen	Mathematical modelling	NA	NA	7 hours	[50]
*Drosophila*	Embryo	Whole body	Perlecan	Mathematical modelling	NA	NA	10 hours	[50]

The table lists the half-lives of basement membrane components reported in literature, the origin of the samples (organism and tissue), the age or body weight of source animals, and the method used to infer the half-lives. The probes used for pulse-chase experiments, and the method for probe delivery are also shown. (*) Silver administered from drinking fluid accumulates in the glomerular basement membrane (GBM). GBM turnover was estimated by measuring the decay of silver level in the GBM after pulse-labelling. (**) The half-life of GBM proteins was calculated from the degree of non-enzymatic glycation of proteins naturally occurring with time. Lower glycation suggests higher turnover. For detail, see ref [81]. (***) This value may not reflect normal turnover because the knockout affected tissue physiology. BW, body weight; GAG, glycosaminoglycan; GBM, glomerular basement membrane; HSPG, heparan sulphate proteoglycan; i.p., intraperitoneal; i.v., intravenous; s.c., subcutaneous.

A recent study supports this idea. There, the overall turnover of basement membrane components in the entire *Drosophila* embryo was measured using a novel method combining live-imaging and mathematical modelling [50]. This study utilized *Drosophila* strains expressing basement membrane components type IV collagen and perlecan that are endogenously labelled with GFP without disrupting protein functions. First, the expression dynamics of type IV collagen and perlecan proteins were measured by quantifying GFP fluorescence in whole embryos. Next, type IV collagen and perlecan mRNA expression dynamics were obtained from the *Drosophila* modENCODE database [82]. Finally, using these protein and mRNA dynamics and a mathematical model that is analogous to other models used to analyse the dynamics of virus or intracellular proteins [83–85], the degradation rates of type IV collagen and perlecan were calculated [50,86]. This revealed remarkably rapid turnover of the basement membrane components (half-life ∼7–10 h), which was further confirmed by *in vivo* pulse-chase experiments [50]. This is consistent with the idea that the embryonic basement membrane should be rapidly turned over to accommodate drastic tissue remodelling.

### 3.3. Basement membrane turnover in the embryo is partially dependent on nidogen and Mmp1

Subsequently, the mechanism of the basement membrane turnover was investigated. Type IV collagen degradation rates in various mutants were calculated using the same mathematical model. First, it was revealed that loss of a basement membrane component nidogen enhanced the degradation about 20%, suggesting that nidogen is protecting type IV collagen from proteolysis. This was consistent with previous reports that nidogen stabilizes the basement membrane [87,88]. No change in type IV collagen level was observed in perlecan (*trol*) mutants, highlighting a specific role of nidogen in regulating type IV collagen stability [50]. Next, it was found that type IV collagen degradation was slowed down about 20% in the absence of Mmp1 [50], one of the two MMPs contained in the fly genome [89]. As Mmp1 accounted for only ~20% of the degradation, other proteases must have also been involved. One candidate was Mmp2, the other *Drosophila* MMP. However, *Mmp2* mutants did not show altered type IV collagen turnover, or *Mmp1-Mmp2* double mutants did not exacerbate the turnover delay of *Mmp1* single mutants [50]. Nevertheless, this does not necessarily mean that Mmp2 is not involved in type IV collagen turnover. Because the modelling calculates the average turnover in the entire embryo, Mmp2ʹs contribution could be missed if this enzyme was responsible for local type IV collagen turnover in restricted tissues (also see section 4.4). Another protease that could be responsible for type IV collagen turnover is AdamTS-A, whose loss and overexpression increases and decreases type IV collagen level, respectively [90]. Analysing basement membrane component degradation rates in the mutants of *AdamTS-A* and other proteases would reveal how multiple proteases are collaborating to ensure proper basement membrane turnover.

### 3.4. Mmp1 enhances the incorporation of type IV collagen to the basement membrane and regulates tissue development and repair

It was previously shown that *Mmp1* mutant larvae harboured less type IV collagen in the basement membrane underneath the epidermis than wild-type animals, while the overall levels of type IV collagen were not reduced by Mmp1 loss. Based on these results, it was suggested that Mmp1 is required to facilitate collagen deposition into the basement membrane [91]. Consistent data were obtained in the embryo: while loss of Mmp1 increased the total amount of type IV collagen, *Mmp1* mutant embryos harboured a reduced level of type IV collagen in the basement membrane surrounding the ventral nerve cord [50]. These results from larvae and embryos suggest that Mmp1 may be essential for proper incorporation of type IV collagen into the basement membranes in many different tissues. In contrast, mutants of another potential type IV collagen-degrading enzyme AdamTS-A accumulate more type IV collagen on the surface of the larval ventral nerve cord than control animals [90]. These results suggest that the role in the incorporation of type IV collagen into matrix is specific to Mmp1.

**Figure 6.**
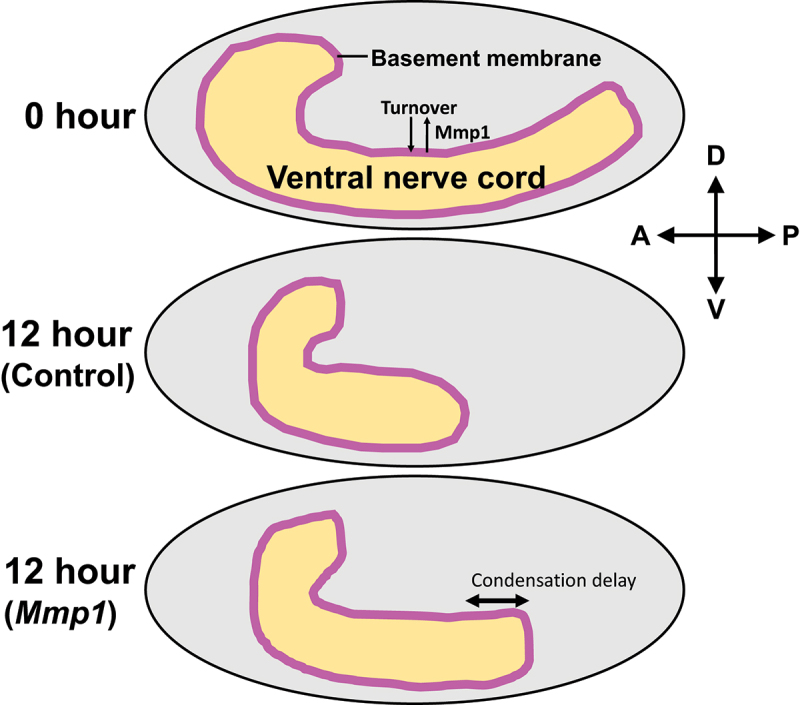
Schematics of the ventral nerve cord condensation during *Drosophila* embryogenesis. (Top) The ventral nerve cord in a stage-15 embryo. The ventral nerve cord is covered by the basement membrane (magenta line) that is rapidly turning over in a manner partially dependent on Mmp1. (Middle) In the control embryo, the nerve cord shortens its length by ~50% in 12 hours. (Bottom) The condensation is delayed in *Mmp1* mutants. A, anterior; P, posterior; D, dorsal; V, ventral.

The functional importance of the Mmp1-mediated type IV collagen turnover/incorporation has also been reported. *Mmp1* mutant larvae are deficient in epidermal wound healing and the assembly of type IV collagen at wound edges [91]. During *Drosophila* embryogenesis, the ventral nerve cord shortens its length by ~50% in a manner dependent on the basement membrane[92,93] (Figure 6). This ventral nerve cord condensation was revealed to be slower in *Mmp1* mutant embryos than in control animals [50]. These results with the epidermis and nerve cord suggest that proper basement membrane turnover is necessary for normal morphogenesis and tissue repair. Mmp1-dependent proteolysis may make the basement membrane pliable enough to allow tissue remodelling. Alternatively, it could also be possible that the reduction of type IV collagen level incorporated in the basement membrane impaired the function of the matrix to support tissue movement.

It is unclear how Mmp1 enhances the incorporation of type IV collagen into the basement membrane. One possibility could be that Mmp1 cleavage of certain basement membrane component(s) exposes new surfaces to which more type IV collagen molecules are recruited. This was hypothesized by an analogy to a regulation mechanism of cytoskeletal dynamics: cofilin-mediated severing of actin filaments produces new free filament ends at which monomeric actin can polymerize, increasing the number of actin molecules incorporated into filaments [94]. High-resolution imaging of basement membrane dynamics such as that with lattice light-sheet microscopy [22] may help examine this ‘surface exposure’ hypothesis. By whatever mechanism Mmp1 enhances the incorporation of type IV collagen into the basement membrane, it is of note that normal bone development and density also depend on constant turnover of ECM by osteoclasts [95]. Therefore, various ECMs require partial degradation for proper development.

Proteolysis-dependent stimulation of type IV collagen incorporation into the basement membrane may also be occurring in *C. elegans*. In the worm, knockdown of a basement membrane protein papilin caused a ‘fibrosis’-like phenotype, i.e. an excess amount of type IV collagen was accumulated on the surface of the gonad. Counterintuitively, this phenotype was suggested to be dependent on the **over**activation of a protease ADAMTS9/GON-1. While knockdown of GON-1 did not phenocopy the fibrosis caused by papilin loss, double knockdown of papilin and GON-1 rescued the phenotype. Moreover, in the absence of papilin, more GON-1 was recruited to the basement membrane. These results suggest that by limiting basement membrane association of GON-1, papilin is restricting matrix degradation by this protease. Therefore, the GON-1-dependent fibrosis of papilin knockdown worms [17] suggests that proteolysis by GON-1 may be enhancing the incorporation of type IV collagen into the basement membrane, similarly to the case with *Drosophila* Mmp1[50,91].

ADAMTS9/GON-1 is homologous to *Drosophila* AdamTS-A, which was suggested to be involved in type IV collagen turnover (section 3.3). Overexpression of AdamTS-A in *Drosophila* did not increase type IV collagen level on ventral nerve cord surface, but did lead to an excess accumulation of another basement membrane component perlecan on the ventral nerve cord [90]. Therefore, ADAMTS family proteases may be regulating basement membrane component incorporation in various organisms.

### 3.5. Baseline turnover of fibrillar matrices

In contrast to the case with the basement membrane, the baseline turnover of fibrillar matrices during embryogenesis has not been quantified. It is intriguing to test whether the extensively moving fibrillar matrices in the early embryo (sections 2.2 and 2.6) [57,58,69] are also turned over, and if so whether the turnover is important for the formation of tissues such as the mesoderm, notochord, and somites.

While the fibrillar matrix turnover in the embryo remains to be explored, the baseline turnover of fibrillar matrix components in postnatal tissues has been investigated in several systems (Table 4). These studies revealed some fundamental features of fibrillar matrix turnover. First, the half-lives of fibrillar collagen are one or more orders of magnitude longer than those of non-collagen fibrillar matrix components (15–215 years vs. 2–25 years) [96–100]. Second, collagen half-lives differ among tissues: in human, 15 years in skin vs. 117 years in cartilage [97]; in horse, 198 years in superficial digital flexor (SDF) tendon vs. 34 years in common digital extensor (CDE) tendon [100]. Third, turnover is affected by age: collagen half-lives in intervertebral discs are longer in older people than in younger people [99]. It remains to be elucidated what determines the half-lives of fibrillar matrix components and what is the physiological and pathological importance of the different turnover.

**Table 4.** Reported half-lives of fibrillar matrix components.

Animal	Age or BW	Tissue	Components examined	Method	Externally added probe	Probe delivery method	Half-life	Ref
Rat	100–115 g	Tail	Entire tail tendon	Pulse-chase	^3^H proline	i.p. injection	>70 d	[2]
Human	55 y	Cartilage	Aggrecan	Asp racemization (*)	NA	NA	3.4–25 y	[96]
Human	Not specified	Cartilage	Collagen	Asp racemization	NA	NA	117 y	[97]
Human	Not specified	Skin	Collagen	Asp racemization	NA	NA	15 y	[97]
Human	0–62 y	Intervertebral discs	Aggrecan	Asp racemization	NA	NA	5.6–22 y	[98]
Human	20–40 y	Intervertebral discs	Collagen	Asp racemization	NA	NA	95 y	[99]
Human	50–80 y	Intervertebral discs	Collagen	Asp racemization	NA	NA	215 y	[99]
Horse	4–30 y	SDF tendon	Collagen	Asp racemization	NA	NA	198 y	[100]
Horse	4–30 y	CDE tendon	Collagen	Asp racemization	NA	NA	34 y	[100]
Horse	4–30 y	SDF tendon	Non-collagen ECM components	Asp racemization	NA	NA	2.2 y	[100]
Horse	4–30 y	CDE tendon	Non-collagen ECM components	Asp racemization	NA	NA	3.5 y	[100]
Human	17–55 y	Achilles tendon	Entire tendon	^14^C bomb-pulse (**)	NA	NA	Too long to measure	[101]

The table lists the half-lives of fibrillar matrix components reported in literature, the origin of the samples (organism and tissue), the age or body weight of source animals, and the method used to infer the half-lives. The probes used for pulse-chase experiments, and the method for probe delivery are also shown. (*) The half-life of matrix proteins was calculated from the degree of the racemization of the aspartic acid (Asp) residues of the proteins. Asp residues are spontaneously and constantly converted from L- to D-form *in vivo*. Lower D/L ratio suggests higher turnover. For detail, see refs [97, 99, 100]. (**) This is a method utilizing the radioactive carbon atoms that were shed into the air by nuclear bomb tests and then naturally incorporated into the ECMs of living organisms through food. ECM turnover was estimated by comparing the concentration of ^14^C in samples and the atmospheric level of ^14^C. For detail, see ref [101]. BW, body weight; CDE, Common digital extensor; i.p., intraperitoneal; SDF, Superficial digital flexor.

### 3.6. Circadian turnover of fibrillar matrices

As in Table 4, the half-lives of fibrillar collagen in tendons and cartilages are on the order of 100 years [97, 99–101], suggesting that virtually no collagen turnover is occurring in these matrices in the time scale of days. However, without daily turnover, ECM components damaged by repeated mechanical loads may not be removed. Moreover, if there is no daily matrix turnover, neither synthesis nor degradation of matrix components would occur in the time scale of days. Nevertheless, neosynthesis of fibrillar collagen within one day is detected in the human Achilles tendon [102].

**Figure 7.**
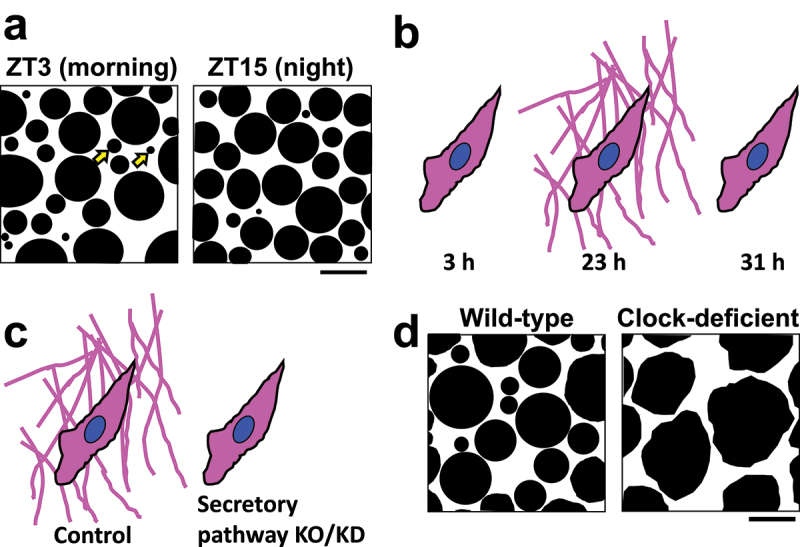
Circadian clock-dependent remodelling of fibrillar collagen. Cartoons are based on the electron (a and d) and fluorescence (b and d) microscope images in ref [103]. (a) Cross section of the mouse Achilles tendon. Each black circle shows a bundle of collagen fibrils. The diameter of the bundles varies, and the number of thin bundles increases at Zeitgeber time (ZT) 3 (in the morning) and decreases at ZT15 (night), respectively. Arrows indicate representative thin bundles. (b) Rhythmical collagen secretion by clock-synchronized cultured fibroblasts. At 3 h after synchronization, type I collagen (magenta) is seen only inside the cells. Subsequently, collagen fibres accumulate in the extracellular space (23 h). In the later phase of the circadian rhythm, extracellular collagen decreases (31 h), suggesting the existence of a clock-regulated degradation step. (c) The knockout (KO) or knockdown (KD) of secretory pathway protein SEC61, TANGO1, PDE4D, or VPS33B inhibits type I collagen fibre assembly in the extracellular space. (d) Cross section of the Achilles tendon of wild-type or circadian clock-deficient mouse. In the mutant mice, the collagen bundles become abnormally thick and irregular shaped. Scale bars, 200 nm.

Recently, it was found that fibrillar collagen molecules are in fact undergoing a remarkable remodelling that is controlled by the circadian clock [103]. In the Achilles tendon, while collagen fibrils form bundles with various diameters, the proportion of thinner bundles was found to increase in the morning and decrease in the night (Figure 7a). To investigate the mechanism of these diurnal changes, the transcriptomic profile of the mouse tendon was examined by microarray. Genes encoding fibrillar collagens did not show circadian oscillations, thus the diameter changes in collagen bundles were not likely to be explained by rhythmic collagen gene transcription. However, genes encoding secretory pathway proteins (SEC61, TANGO1, PDE4D, and VPS33B) and a protease cathepsin K (CTSK), which was previously known to play key roles in collagen turnover in bones and other tissues [39,40,77,95], were found to express rhythmically. Moreover, western blot revealed the oscillating expression of these secretory proteins and protease in the tendon. Their functions were investigated by *in vitro* and *ex vivo* experiments. As to the secretory pathway proteins, their knockout/knockdown impaired the rhythmical increase of fibrillar collagen in the space outside cultured fibroblasts (Figure 7b, c). Regarding CTSK, its chemical inhibitor odanacatib caused collagen accumulation in tendons cultured *ex vivo*. From these results, it was proposed that the rhythmic boost of secretion and degradation by the diurnal expression of the secretory proteins and CTSK protease, respectively, mediate the oscillation of the levels of fibrillar collagen protein in the extracellular space. A mathematical model supported this idea. Furthermore, deficiencies of the circadian clock made the structure of collagen bundles abnormal (Figure 7d), and reduced the elasticity and strength of the tendon. These data indicate that fibrillar matrices are remodelled and turned over every day in a manner dependent on the circadian clock, and suggest that the rhythmic secretion and degradation of fibrillar collagen may underlie the diurnal structural changes seen in the Achilles tendon (Figure 7a).

However, these results seem to disagree with the previous reports that collagen does not turnover daily [97, 99–101]. To solve this problem, it was proposed that there exist a large amount of ‘persistent’ collagen and a small pool of ‘sacrificial’ collagen, and the latter is undergoing the circadian turnover [103]. It remains to be elucidated exactly how much of collagen molecules are ‘persistent’ and ‘sacrificial’ *in vivo*. Classical work in the 1960s suggested that there exist two pools of tadpole tail collagen: the ‘hot’ fibrils that are spared from degradation and the ‘cold’ ones that are preferably broken down during metamorphosis [78]. Whether the ‘hot’-‘cold’ and the ‘persistent’-‘sacrificial’ pools are determined by a similar (or even the same) mechanism is another question to be explored.

Disruption of the circadian clock leads to a number of abnormalities in connective tissues, e.g. the fibrosis, calcification, and degeneration of cartilages and tendons [104–107]. Failure in the circadian turnover of fibrillar matrices may underlie these diverse pathologies [103]. It is unknown why fibrillar matrices should undergo a circadian turnover. One possibility is that the turnover is to adapt to the diurnal changes of mechanical loads [108]. For example, in the mouse intervertebral discs, the expression of genes that are potentially involved in ECM turnover (e.g. those coding ADAM17, ADAMTS1, and ADAMTS15 proteases in Table 5) peaks during waking hours [105]. The products of these genes may remove ECM molecules that are damaged by mechanical loads during waking time, and impaired ECMs may be repaired during sleep.

**Table 5.** Enzymes that are suggested to be involved in the baseline turnover of ECMs.

Enzyme	Animal	Description	Section	Ref
Mmp1	*Drosophila*	*Mmp1* mutants show ~20% slower turnover of type IV collagen compared to control.	3.3	[50]
CTSK	Mouse	A CTSK inhibitor odanacatib causes collagen accumulation in cultured tendon.	3.6	[103]
AdamTS-A	*Drosophila*	Its loss and overexpression increases and decreases type IV collagen level, respectively.	3.3	[90]
ADAM17	Mouse	Expression level oscillates with a 24-hour rhythm in the intervertebral discs.	3.6	[105]
ADAMTS1	Mouse	Expression level oscillates with a 24-hour rhythm in the intervertebral discs.	3.6	[105]
ADAMTS15	Mouse	Expression level oscillates with a 24-hour rhythm in the intervertebral discs.	3.6	[105]

## 4. Conclusion and perspective

The results reviewed above demonstrate that ECMs are dynamically moving and turned over during tissue development and homoeostasis, in contrast to the previously believed idea that ECMs are static structures. However, ECM dynamics *in vivo* have not been fully investigated yet, let alone the molecular mechanisms and functional importance of the dynamics. The following sections discuss remaining questions regarding ECM dynamics, and potential approaches to address them.

### 4.1. What ECMs move; for what and how?

ECM movement has been documented only in a limited number of cases. Relative motion of cells and ECMs is even more unexplored. In the animal body, a countless number of events that involve cell migration occur, e.g. angiogenesis, wound healing, migration of differentiated cells out of stem cell niche, and tumour metastasis. It would be interesting to investigate whether ECMs are also moving during these events, and if so, how the cells are moving relative to the mobile ECMs, and what is the functional importance of the relative motion.

One ECM function that could be affected by the relative movement of cells and matrix is signalling (section 1.2). For example, assume a group of cells migrating on an ECM that contains a gradient of a signalling factor (Figure 2g). If the ECM is not moving, the cells are moving towards a higher concentration of the fixed signalling factor. However, if the ECM is moving with the cells as in the cases with the avian epiblast basement membrane or hydra mesogloea (section 2.6) [62,63], during the migration each moving cell is exposed to a constant level of the signalling molecule. More investigations are needed to understand how moving ECMs *in vivo* signal to the cells in/on them [62].

Generally, to reveal the functional importance of ECM movement in a certain event, it needs to be tested how the event is affected when the movement is impaired; to impair the movement its mechanism should be known. However, as discussed in the next section, knowledge about the mechanisms of ECM motion is currently limited.

### 4.2. The mechanisms of ECM propulsion are largely unknown – can ECMs move cells?

ATP, muscle contraction, proteolysis, and non-muscle myosin activity have been suggested to be necessary for ECM movements (sections 2.3 and 2.4) [17,64]. However, it is still unclear exactly how these factors move ECM components and contribute to tissue development and function. Moreover, when cells and ECMs migrate together as seen in the avian embryo and hydra (section 2.6) [62,63], it cannot readily be distinguished which is moving which. Although cells can exert forces to relocate and remodel ECM [109], it is unclear whether ECMs are always passively locomoted by cells. For example, during the gastrulation of the avian embryo, while cell intercalation occurring in the epiblast [110] could produce the force to move the basement membrane under the epithelium, it may also be possible that the physical and chemical reactions occurring within the basement membrane exert compression or stretching forces to move the epithelium and basement membrane together [62]. Indeed, basement membranes isolated from various human tissues consistently rolled up in one direction, with the epithelial side facing outward and the stromal side inward, independently of the curvature of source tissues [111]. This suggests that intact basement membranes may be continuously exerting elastic forces to tissues. Moreover, when a collagen gel was put next to another gel containing collagen and fibronectin, inert beads contained in the former gel were translocated to the latter, suggesting that non-uniform ECM can generate forces to move the beads *in vitro* [112, 113]. Furthermore, a recent preprint proposed that the ventral nerve cord condensation in the *Drosophila* embryo (**section 3.4**) is at least partially propelled by a sudden increase in ECM-driven surface tension caused by type IV collagen assembly [114]. These results suggest that it is possible that ECMs move cells and tissues.

To understand the mechanism of ECM propulsion, it is necessary (1) to identify biomechanical forces produced by cells and ECMs, e.g. those generated by cell movement and ECM assembly/breakdown, and (2) to reveal the material properties, e.g. elasticity and plasticity, of the cells and ECM that define their responses to the forces in (1) [62]. Recently, new technologies such as high-resolution live imaging, atomic force microscopy, and computational/mathematical modelling/simulation are rapidly revealing the mechanical properties of cells and ECMs [115–125]. These advanced methods will help researchers to investigate the mechanisms of ECM propulsion and their functional importance in tissue development, homoeostasis, and repair [126].

It might be doubted that a ‘lifeless’ polymer network like the basement membrane or any ECM can produce tissue-remodelling forces. However, there is a prominent example of force-generating polymers – cytoskeleton: actin filaments and microtubules propel organelles, chromosomes, and cells by dynamically growing, collapsing, and deforming [127–133]. Dynamic remodelling of ECMs may also drive tissue rearrangements. Indeed, inhibition of proteolysis impaired the branching of salivary gland epithelial tubules [64]; *Mmp1* mutant flies showed defects in ventral nerve cord condensation and wound healing [50,91]; in hydra, mesogloea movement and turnover were reported to be correlated: the body column showed a higher activity to degrade a mesogloea component laminin than the head, while mesogloea was moving in the former but not in the latter [63]. It is interesting to investigate how ECM turnover affects matrix plasticity, whether the forces generated by the addition and removal of components to/from ECMs are strong enough to move tissues, and how ECM remodelling and the rearrangements of cell-cell and cell-ECM adhesion like those recently reported in the developing salivary gland [123] are combined to sculpt tissues. These investigations will reveal the link between the two aspects of ECM dynamics reviewed in this article: movement and turnover.

### 4.3. Mechanisms of the baseline turnover of ECMs

Currently, knowledge about the mechanisms of baseline ECM turnover is limited. Only two proteases responsible for this process have been identified: *Drosophila* Mmp1 for basement membrane type IV collagen turnover and mouse CTSK for the circadian degradation of fibrillar collagen (Table 5). However, for example Mmp1 explains only ~20% of total type IV collagen turnover (section 3.3); therefore, there must be (potentially many) other enzymes involved, e.g. the ADAM and ADAMTS proteases listed in Table 5. As mentioned in section 3.1, there is an enormous accumulation of knowledge about proteases working in other contexts [39,40,70,72–77]. These enzymes and their already known regulators may also be involved in the baseline turnover of ECMs, similarly to the case with Mmp1 that regulates not only baseline basement membrane turnover but also the acute breakdown of the basement membrane surrounding imaginal discs during metamorphosis [134]. The already accumulated knowledge about proteases will boost the investigation of baseline ECM turnover.

Another question about baseline ECM turnover is the destiny of degraded ECM components. It is unknown whether they are completely digested and recycled by local cells, or partially cleaved ECM components are removed from matrix and digested somewhere far away. As to the latter possibility, for example mammalian intervertebral discs are known to routinely experience compressive pressures that squeeze out about 10–25% of total water in the tissue [135–137]; these compressions may flush cleaved ECM components out of the discs [108]. In *Drosophila*, nephrocytes tethered to the oesophagus and the heart take up and degrade proteins that are circulating in the haemolymph (blood) [138–141]; removal of nephrocytes was reported to lead to the abnormal accumulation of ECM proteins SPARC and perlecan in the haemolymph [142]. These results suggest that nephrocytes are responsible for the final degradation of ECM components. Thus, turnover is part of the ‘life-cycle’ of ECM components, in which matrix proteins are synthesized, assembled, cleaved, carried to final degradation site, and completely digested.

### 4.4. Mathematical modelling as a potential tool to interrogate the turnover of various ECMs

When analysing the role of many proteases or different factors in baseline ECM turnover, a simple method to measure the turnover in various backgrounds is required to enhance research efficiency. Recently, a novel method based on live imaging and mathematical modelling was designed and used to reveal the rapid turnover of basement membrane components in the *Drosophila* embryo (section 3.2)[50]. The advantages, limits, and potential applications of this new approach are discussed below.

One of the advantages of the method is simplicity: measurements can be done with standard fluorescence dissection microscope and an uncomplicated mathematical model. The expression dynamics of type IV collagen and perlecan endogenously labelled with GFP were measured in whole embryos without dissection/purification of target tissues/molecules.

However, this simplicity is not only an advantage but also a limitation of the method: while the turnover calculated here shows the average protein degradation rate in the entire body, local difference in basement membrane turnover cannot be measured. Thus, the potential role of Mmp2 in local type IV collagen turnover may have been missed (section 3.3). Moreover, basement membranes in different tissues contain distinct sets of components and play location-specific roles (section 1.2). Therefore, it is interesting to examine whether basement membrane turnover rates are also different among tissues and whether the turnover difference is related to the specific functions of each basement membrane. To analyse local basement membrane turnover, researchers need a more elaborate imaging system and a new mathematical model that takes into account the movement of basement membrane components into and out of the area of interest.

Care should also be taken about the maturation and quenching of the fluorophore used for live imaging. In the case of the work in **section 3.2** [50], type IV collagen and perlecan were tagged with GFP. The maturation time of GFP fluorophore is relatively fast (~14–60 min after protein folding) [143] compared to the time it took for the fluorescence to reach homoeostasis from initial induction (> 12 hours). Thus, the time lag between protein folding and fluorophore maturation should have caused only a minor effect on the modelled expression dynamics of the tagged proteins. As to quenching, it was shown that GFP was not photobleached during measurements [50]. However, the effect of bleaching should be considered when stronger excitation or an easily bleaching probe is used in future. Even not being photobleached, GFP also has its own lifetime. The half-life of GFP itself was reported to be ~26 hours in cultured mammalian cells [144]. It is three times as long as the reported half-lives of basement membrane components [50]; thus, it is unlikely that the GFP half-life affected the measurement of the turnover of basement membrane. However, GFP would not be a suitable probe to measure the turnover of proteins with a longer half-life.

Another advantage of the modelling-based method [50] is its intervention-free nature: no invasive actions such as probe injection are required. There are several other intervention-free methods to measure ECM component turnover. They utilize e.g. 1) naturally accumulating glycation of ECM components [81], 2) naturally accumulating chirality changes (racemization) of amino acid residues in ECM components [97, 99, 100], or 3) radioactive carbon incorporated into ECMs from the environment [101] (Tables 3 and 4). However, these methods have some caveats. As to the glycation-based calculation, precise measurement can be hampered by the fluctuation of ECM glycation rates caused by anything that affects the sugar level in the body fluid: e.g. starvation and diabetes. As to the approaches based on amino acid racemization and radioactive carbon, they can detect only slow turnover that occurs on the time scale of years or more. In contrast, the mathematical modelling can infer turnover occurring at any rate and in any pathophysiological background, if the temporal changes of the protein and mRNA levels are measured and the two following simple assumptions stand: 1) protein synthesis is proportional to the level of mRNA coding the protein, and 2) protein degradation (turnover) is proportional to the amount of the protein itself. These prerequisites are suggested to be true for many proteins, e.g. for about one-third of all the proteins in yeast [145]. Therefore, the model would be useful to infer the turnover rates of not only *Drosophila* type IV collagen and perlecan but also many different proteins in flies and other systems such as worms, fish, and cultured cells/tissues. Many transgenic animals/cells that express fluorescent ECM components that could be suitable for the modelling are already available [17,146–148], and a detailed protocol has also been published [86].

### 4.5. Conclusion

As reviewed above, ECM dynamics is now emerging as a hitherto unrecognized critical factor for tissue development and maintenance. Powerful new methods such as genome editing, live imaging, and mathematical modelling are helping researchers to address fundamental questions about ECM dynamics and its mechanisms and functions. An exciting time in matrix biology research is now starting.

## Data Availability

Data sharing is not applicable to this article as no new data were created or analysed.
